# Error monitoring and daily life executive functioning

**DOI:** 10.1007/s00221-019-05589-w

**Published:** 2019-06-24

**Authors:** Saleh M. H. Mohamed, Norbert A. Börger, Reint H. Geuze, Jaap J. van der Meere

**Affiliations:** 1 Department of Clinical and Developmental Neuropsychology, Faculty of Behavioural and Social Sciences, University of Groningen, Grote Kruisstraat 2/1, 9712 TS Groningen, The Netherlands; 2 Department of Psychology, Beni-Suef University, Beni Suef, Egypt

**Keywords:** Post-error slowing, Executive functions, Ecological validity, Daily functioning, Academic achievement

## Abstract

Error monitoring during task execution is reflected in post-error slowing (PES), which refers to the tendency to slow down performance after making an error in order to prevent future mistakes. The key question of the present study is whether poor error monitoring (reduced magnitude of PES) has negative consequences for daily life executive function skills, as well as functioning in different life settings such as work, family, social, and academic settings. Eighty-five university students performed a lexical decision task and completed The Executive Function Index Scale (EFI), and the Weiss Functional Impairments Rating Scale (WFIRS). Individual academic achievement was measured using the Grade Point Average. Statistical analysis revealed that a decreased magnitude of PES was weakly associated with less efficient planning (one of the executive functions). Results suggest that error monitoring, as measured by PES, was not associated with functioning in a naturalistic environment, but could be interpreted to some extent as an experimental marker of planning in daily life executive functioning.

## Introduction

Post-error slowing (PES) is a well-known phenomenon: when an error has been made, and the individual is aware of it, performance slows down to avoid a subsequent error (Gruendler et al. 2011; Notebaert et al. 2009). PES has been considered an outcome of how our performance monitoring system works which compares the actual executed response with the required (target) response (Gruendler et al. 2011). The detection of a mismatch between an actual response and the required response is thought to enable us to be more careful, as well as to take more time in completing information processing and/or preparing for the appropriate motor response. From this strategic-evaluation account, one might assume that a standard interpretation of PES is that individuals actively (by means of strategy) evaluate their own performance and adjust their speed-accuracy balance towards a more conservative criterion whenever they detect an erroneous response (Steinborn et al. 2012). Hence, the error is construed as having positive effects on subsequent performance, yielding more attentive, slower and more accurate performance in subsequent trials. However, this is not the only account to explain the PES. Another well-known explanation of PES is the orienting account, which argues that when errors are infrequent events, errors function to orient participant’s attention towards unexpected task-irrelevant signals (Notebaert et al. 2009). According to this perspective, an error is thought to have negative effects on subsequent performance, such as increasing arousal, disrupting subsequent information processing, and resulting in slower and less accurate performance in the subsequent trials. In sum, from the orienting account, post-error slowing and accuracy decline is more pronounced, while from the strategic-evaluation account there is no such assumption about performance decline. Alternative explanations have been proposed for PES; for instance, PES is explained by automatic response inhibition after errors (Gupta et al. 2009), or that the deficient process that causes an error may last, causing slow and incorrect response on a subsequent trial (see, Notebaert et al. 2009). In these latter accounts, awareness of errors does not necessarily lead to PES. Klein et al. (2007) discussed that PES seems to interact with and to be modulated by error awareness. They found that PES is only present for the perceived errors relative to the unperceived errors and speculated “the error signal needs to exceed a certain strength or signal-to-noise ratio to allow the conscious perception of the error and subsequent adjustments”. This may explain why PES is more likely to be present when errors are consciously perceived.

Many of the abovementioned accounts of PES have been considered for further investigation using a large set of lexical decision data and other types of tasks such as the flanker task, the Go/No-Go task, and multiple-choice arithmetic tasks. Overall, the results of these investigations support the notion that PES reflects cognitive control and performance monitoring rather than other PES accounts such as the orienting account (Dutilh et al. 2012a; Lavro et al. 2018; Wang et al. 2016).

Although PES has various interpretations, the phenomenon has been studied in a variety of patient groups such as attention-deficit/hyperactivity disorder (for a meta-analysis see Balogh and Czobor 2016; Czobor et al. 2017), Autism (for review see Rommelse et al. 2011; Sokhadze et al. 2012), and Schizophrenia (Kerns et al. 2005; Moran et al. 2018). Studies showed that these patients do not exhibit a slowing of performance after committing an error, or at least patients demonstrated decreased PES and less pronounced electrical brain potentials associated with error processing. In most of these studies, comparing normal controls with patients, the error rate was more pronounced in the patient groups relative to the controls, which could be the actual cause of the smaller (or even absent) PES effect in the patient groups. Findings here show that decreased PES or its absence is related to several dysfunctions and atypical behaviors. As important as these laboratory findings are, very little is known about the negative consequences of having a lesser PES, as assessed during an experimental task, on everyday functioning in healthy populations. So far, the ecological validity of the PES in healthy populations has been studied in relation to the daily coping with stress (Compton et al. 2011) and academic achievement (Fisher et al. 2009; Hirsh and Inzlicht 2010). These studies highlight the negative functional consequences of having a lesser magnitude of PES, such as low academic achievement and being less able to control emotional reactions towards daily stressors.

The present study, to the best of our knowledge, is the first to explore the ecological validity of the PES from the perspective of the quality of executive functions in daily life, as well as functioning in different settings of life (such as work, family, and social settings). The study is motivated by the fact that error monitoring (error detection and response adjustments after errors) is a critical executive function for flexible interaction with changing environmental conditions (Kim et al. 2016; Garavan et al. 2002), and thus essential for learning and self-regulation (for review see, Aarts and Pourtois 2015). Error monitoring also plays an important role in other executive functions, experimental studies have shown that task indices of low monitoring can predict poor performance on tasks measuring working memory and response inhibition (see Robinson et al. 2010; Silver and Goodman 2007; Silver et al. 2006). From the brain activity perspective, some EEG studies reported associations between electrophysiological error-related brain potentials (i.e. ERN/CRN and Pe amplitude) and performance on some executive functions tasks such as Go/no-Go tasks (Grammer et al. 2017; Kim et al. 2018).

One motivation to choose for PES as correlate of error monitoring instead of other post-error adjustments, is that several neuroimaging studies show an association between PES and the posterior medial frontal cortex activity, while other post-error response adjustments, such as post-error response accuracy and post-error reduction of interference, are associated with activities in task-related visual brain areas (Danielmeier and Ullsperger 2011). The neuroanatomical basis of executive functioning is also suggested to be located in the posterior medial frontal cortex (see Black et al. 2011). It may worth noting that executive functions refer to a family of top-down cognitive control processes (Diamond 2013). It is found that increasing cognitive control demands increase PES in particular (see Regev and Meiran 2014). Another motivation of choosing PES is that PES seems reliable on a within-subject level over a long period of several months and independent from post-error response accuracy and post-error reduction of interference, however, PES might facilitate other post-error adjustments (Danielmeier and Ullsperger 2011).

In the present study, a sample of university students is tested on their error-monitoring ability (indexed by post-error slowing) and their daily executive functioning. This allows examining the continuous relationship between varying levels of executive functions and the magnitude of PES in a nonclinical population that received very little attention in the literature. In such a sample, error processing and related compensatory behavioral adjustments contribute to the academic progress (Fisher et al. 2009; Hirsh and Inzlicht 2010), and success in social and occupational lives. One can utilize error monitoring in managing responses, thoughts, and feelings, leading to good functioning in social and occupational lives (McClelland and Cameron 2011; McClelland et al. 2018; Moser et al. 2011). Studies show that a greater magnitude of PES is associated with better stress regulation, and a higher level of well-being and life satisfaction (Compton et al. 2011; see Robinson et al. 2010).

To this end, university students performed a lexical decision task from which the magnitude of PES is calculated and correlated with total scores on two self-report scales: the Executive Function Index scale (EFI; Spinella 2005) and the Weiss Functional Impairments Rating Scales (WFIRS; Weiss 2010). In addition, the correlation between the magnitude of PES and academic achievement scores (as estimated by Grade Point Average; GPA) is tested. Based on the literature, the hypothesis is that decreased magnitudes of PES are associated with (1) lower total scores on the EFI, (2) higher total scores on the WFIRS, and (3) lower GPA scores.

To gain more insight into which specific executive function and which specific daily life settings are associated with error monitoring, the correlations of PES with scores on the subscales of EFI and WFIRS are tested. Having said that, it can be argued that everyday functioning (measured by WFIRS subscales) and executive functions in daily life context (measured by the EFI subscales) are very close to each other; making it difficult to differentiate between them in their association with PES. To test if the EFI and WFIRS are dependent on each other and reflect the same constructs, correlations between the subscales of the EFI and WFIRS are tested.

## Method

### Participants

Eighty-five students (43 females) of the University of Groningen were recruited. The mean age was 21.5 years (SD 2.9), ranging from 18 to 31 years. The Ethics Committee Psychology of the University of Groningen approved the study. In addition, informed consent was obtained from the participants.

### Measures and apparatus

#### Lexical decision reaction time task

Participants were given a lexical decision task used in our previous work (see Mohamed et al. 2016). The task was designed using E-prime software version 2.0. The task had two versions: one in Dutch language and the other one in German language.

Each trial in the task started with a fixation cross displayed for 200 ms on a laptop screen, followed by a stimulus (two letter strings), which were presented for 150 ms to the left and right of the fixation cross position. The distance between the two letter strings was 2.46° of viewing angle. The inter-stimulus-interval was 4000 ms during which the fixation cross was presented, and wherein participants could give their responses.

Each stimulus had two letter strings presented at the same invisible horizontal line: one letter string was underlined (the target) and the other one was not (the distractor). Displaying the distractor together with the target simultaneously aimed to increase the level of task difficulty. Each letter string (either a meaningful word or a pronounceable meaningless non-word) consisted of three, four, or five letters. In each stimulus, the target and the distractor had an equal number of letters. Half of the target letter strings were words and the other half were non-words. There were four possible combinations between the target and the distractor in each stimulus: word (target) with word (distractor), word (target) with non-word (distractor), non-word (target) with word (distractor), or non-word (target) with non-word (distractor). Non-words were generated by Wuggy software (Keuleers and Brysbaert 2010) and Dutch and German words were selected from CELEX (Baayen et al. 1995) and SUBTLEX (Brysbaert et al. 2011) databases. Six native speakers (three were German and three were Dutch) reviewed the selected list of Dutch and German words and their corresponding pronounceable non-words based on their native language. The native speakers were also asked to indicate whether the combination between two presented letter strings (the target and the distractor) at one time could be perceived as a word. Based on the native speakers’ revisions, a list of letter strings (words and non-words) was selected to be used as task stimuli. The goal of having such a procedure is to make sure that errors are not related to the way the two letter strings were displayed. The task consisted of four blocks of 192 trials. In each block, the four combinations of the target with the distractor were counterbalanced and presented in a random order without repetition. There were three breaks between the blocks; each break was about one and half minutes, so the eyes of the participants could have a rest, making testing procedures more comfortable for participants. The task duration was about 15 min.

Each participant received verbal and written instructions, asking them to decide as quickly and as accurately as possible whether the target (underlined) letter string is a word or non-word based to participant’s native language (Dutch or German). The decision was made by pressing one of two different buttons on a response box (i.e. a button on the left side of the response box was assigned for words and the other one on the right side was assigned for non-words). No feedback was given after a correct or an incorrect response. Although task instruction put relatively equal emphasis on fast responding and high accuracy, given this instruction with fast stimulus presentation (150 ms) and the absence of feedback it may be assumed that the testing situation might make participants more oriented to fast responding rather than high-performance accuracy. It has been shown that rapid stimulus presentation over a block of trials may arouse participants to react as fast as possible (see Christ 1970). This, in turn, might give the participants less chance to improve their responses after errors.

The lexical decision task has a long tradition in investigating post-error behavioral adaptations and error processing (see for instance Dutilh et al. 2012a; Kaplan and Zaidel 2001). Conclusions are often drawn from these studies about individual potentials to adapt and monitor performance or/and to process errors. In this vein, we used a lexical decision task aiming to test whether these individual potentials and their underlying mechanisms are directly associated with daily functioning.

#### Executive Function Index Scale

The Executive Function Index Scale (EFI) was used to measure executive functions in daily life contexts. The scale consisted of 27 items constituting five scales: Motivational Drive, Organization, Impulse Control, Empathy, and Strategic Planning. The Motivational Drive scale taps behavioral drive and interest in novelty. The Organization scale measures organizational skills such as multitasking, sequencing, and keeping things in mind. The Impulse Control scale considers inhibition ability. The Empathy scale addresses the tendency to behave in a prosocial way. One might question how empathy is an executive function. In this regard, Rueda and Paz-Alonso (2013) discussed that empathy is essential for managing emotions in social interactions, and specifically in setting goals accommodated to social norms, planning and executing goal-directed behaviors in social context. Furthermore, several studies showed a subtle connection between empathy, as a component of theory of mind (ToM) and executive functioning at both the behavioral and the brain activity levels (Bertoux et al. 2016; for reviews see also Decety and Jackson 2004; Decety and Jackson 2006; Eslinger et al. 2011; Jolliffe and Farrington 2004).

Finally, the Strategic Planning scale assesses planning and thinking ahead, and the tendency to use strategies (Spinella 2005). Subjects rate themselves on a 5-point Likert scale (scored from 1 = not at all to 5 = very much). Scores on 12 items, distributed over the entire scale, were reversed, and a higher total score of the EFI scale indicates better executive functioning in daily life context (Spinella 2005). The scale has shown adequate validity with strong correlations with other self-rating executive functioning scales validated in clinical studies using the Frontal Systems Behavior Scale, and neuroimaging studies (Miley and Spinella 2006; Spinella 2005). The EFI demonstrates good internal consistency: the total score showed good reliability (Cronbach’s alpha = 0.82) as well as the five subscales (Cronbach’s alpha across the five subscales ranged from 0.69 to 0.76) (Spinella 2005). In contrast to many of the existing executive function scales, the EFI scale was not mainly created for clinical purposes. The scale was developed for a community sample recruited from the college campus and the local community, making it more suitable for the sample of the present study to measure the level of executive functions skills. Overall, there is a lack of studies showing the ecological validity of the EFI for everyday executive functions in healthy populations. A few studies showed the scale has good ecological validity: scores on the scale were significant predictors of scores on other scales such as the Motivated Strategies for Learning Questionnaire (MSLQ) measuring everyday behaviors that reflect the use of cognitive and metacognitive strategies (e.g. self-monitoring and planning), and academic effort regulation in college students (Garner 2009). A recent study by Lin et al. (2018) showed that the EFI scale, as a measure of executive functions, was related to critical and creative thinking. The scale was used to study ADHD and autistic traits in the non-clinical population (Ferraro et al. 2018; Mohamed et al. 2016). However, to the best of our knowledge, no study has directly tested the association between the EFI and external markers of executive functions in a healthy population. There are several studies, however, that showed the EFI is fit to be used in healthy populations, capturing enough variance, see for example Kruger (2011) and Weatherly and Ferraro (2011), the variance of the scales in these studies is in line with our study.

#### Weiss Functional Impairment Rating Scale (WFIRS)

The WFIRS measures adult’s functional impairments. The scale consists of 70 items distributed over seven scales: Family, Work, Learning/College, Life Skills, Self-Concept, Social Functioning, and Risk Taking. Items represent problems in everyday situations. Each item was measured on a four-point scale (scored from 0 = never or not at all to 3 = very often or very much). In case some items were not applicable to the participant, the participant could response with ‘not applicable’. For each scale, the average score was calculated as the sum score of the responses on the scale divided by the number of responses. The total score of the WFIRS was calculated by summing the average scores of all scales (NACE 2014; Weiss 2010). Higher scores indicate an increased number of functional impairments and low scores indicate better functioning in daily life activities.

The scale has good internal reliability and offers adequate convergent, discriminate, and concurrent validity (see Canu et al. 2016; Gajria et al. 2015); for example, Canu et al. (2016) created a collateral version of the scale and found strong internal consistency. In addition, the WFIRS scale showed strong relationships with other well-established scales of functional impairments in student population, namely the Current Symptom Scale and the Pediatric Quality of Life Inventory (Hadianfard et al. 2017). The scale also showed moderate convergent validity (*r* = 0.6) with other measures of functioning, namely the Columbia Impairment Scale and the Global Assessment of Functioning Scale (Takeda et al. 2017).

Although the WFIRS has been considered specific to ADHD as it was developed for the ADHD population, previous validation studies have shown that the measure has good psychometric properties in a normal population (see, Weiss et al. 2018). For instance, the study of Canu et al. (2016) demonstrates the WFIRS captures enough variance in university students, which is comparable to the variance in our study. Mean scores and standard deviation of scores on all scales of the EFI and the WFIRS of our study sample are presented in Table 1.

**Table 1:** Means, standard deviations, and skewness of scores on all scales of the EFI and the WFIRS, performance measures, and GPA

Scales and RT measures	MD	ORG	IC	EM	SP	EFI Total score	Family	Work	College	Life Skills	Self-concept	Social Functioning	Taking Risk	WFIRS Total score	GPA (*N* = 56)	RT	Error rate	SD of RT	PES robust	PES Traditional	PCA	PEA
*M*	14.43	15.74	17.07	24.49	21.56	93.27	0.65	0.47	0.75	0.85	0.99	0.52	0.56	4.81	6.92	785	17.21	197	23.57	7.44	83.16	85.74
SD	2.58	3.82	3.46	3.60	5.76	11.70	0.50	0.60	0.62	0.60	0.81	0.46	0.43	3.09	1.10	112	8.77	55	51.18	49.56	7.93	10.81
Skewness	− 0.22	0.02	− 0.28	− 1.21	− 0.09	− 0.43	0.95	1.39	0.94	0.51	0.49	1.15	1.18	0.80	− 0.43	0.39	1.21	0.42	0.18	0.56	− 0.69	− 1.22

The number of participants for all variables was 85, except for the GPA it was 56

*EFI* Executive Function Index scale. The subscales of the EFI are: *MD* motivational drive, *ORG* organization, *IC* impulse control, *EM* empathy, *SP* strategic planning, *WFIRS* Weiss Functional Impairment Rating Scale. The subscales of the WFIRS are: family; work; college; life skills; self-concept; social functioning. *RT* mean reaction time of correct responses, *SD of RT* standard deviation of mean reaction time of correct responses, *PES robust* the robust measure of post-error slowing, *PES traditional* the traditional measure of post-error slowing, *PCA* post-correct accuracy rate, *PEA* post-error accuracy rate, *GPA* grade point average. Standard error of skewness for GPA was 0.319, standard error of skewness for all other variables was 0.261

#### Grade point average (GPA)

Grade point average scores were taken to reflect the academic achievement of the participants. GPA score was individually calculated by dividing the total magnitude of earned grade points by the total number of grade points of that year. Only 56 students gave the authors permissions to collect their academic achievement data at the end of the academic year.

### Procedure

The study had two testing sessions. In the first session, participants were asked to fill out a number of questionnaires, which lasted about 45 min on average. In the second session, participants performed only a reaction time lexical decision task. In both sessions, participants received written information about the study and their anticipated tasks. Participants agreed and signed an informed consent before their participation; thereafter, they received instructions. At the beginning of the first session, the researcher gave verbal instructions to answer the paper and pencil questionnaires as thoroughly as possible and to report essential information such as age, and gender on each printed questionnaire. Participants were also instructed to read carefully and follow the written instructions of each questionnaire before start answering it. At the end of the first session, participants were asked to check whether they missed one or more items and to address the missing items. During the session, participants filled out the following questionnaires: the EFI Scale, the WFIRS Scale, the Boredom Proneness Scale, and the Depression, Anxiety, and Stress scale. Please note only data from the EFI, and the WFIRS scales were used in statistical analyses because these were of actual interest in the present study. These questionnaires were filled out in different times: some at the same day of the experimental session others before the experimental session.

For the second session, written task instructions were presented twice: at the beginning of the task and a second time before the third block in order to refresh and remind the participants with proper responses needed to complete the task successfully. Using a response box, the instructions were to press a specific button labeled “1” if the presented target letter strings is a word and to press a different button labeled “2” when it is a non-word. Participants received practice trials before performing the four task blocks. The examiner gave also general oral instructions, as follows: “A fast and correct response is required on all task trials and does not miss any of the trials”. Using a response box, the instruction was to press a specific button labeled “1” if the presented target letter strings is a word and to press a different button labeled “2” when it is a non-word. Participants received practice trials before performing the four task blocks. At the end of the second session, participants received a debrief sheet, wherein the goal of the study and expectations were explained in detail. During this experimental session, the Edinburgh Handedness Inventory was administrated, data about handedness was collected for other research.

### Data analysis

Mean reaction times (RT) of correct responses and those of errors were calculated separately for each participant. Responses faster than 200 ms were excluded from calculating mean RT of correct responses (please note. responses faster than 200 ms were overall few in average around three trials). Classifying a non-word as a word or vice versa was counted as an error. Standard deviation of mean RT of correct responses was calculated. Error rate was measured using the following formula: error rate = [(number of incorrect responses/the total number of trials) × 100].

Accuracy rate after correct responses (PCA) and that after errors (PEA) was separately calculated using the following formulas: PCA = [(number of correct responses after correct response/the total number of correct responses) × 100]; PEA = [(number of correct responses after errors/the total number of errors) × 100].

**Fig. 1:**
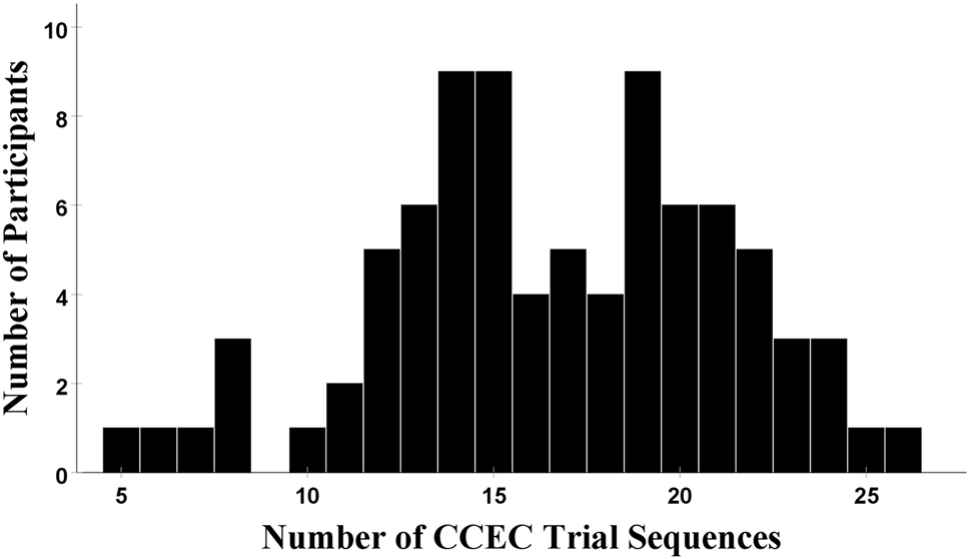
The distribution of participants/number of CCEC trial sequences; wherein the pre-error trial was a correct response that followed a correct response, and the post-error trial was a correct response as well

For each participant, the magnitude of PES was quantified using two measures: the traditional measure and the robust measure (see, Dutilh et al. 2012b). The traditional measure of PES is the difference in mean RT between post-error and post-correct responses. The robust measure of PES is the mean difference between RT of pre-error trial (Error − 1) and RT of post-error trial (Error + 1) for trial sequences, wherein pre-error trials were correct responses that followed correct responses and post-error trials were correct responses (i.e. Correct_(E−2)_ → Correct_(E−1)_ → Error_(E)_ → Correct_(E+1)_). Whether these trial sequences were followed by correct trials was not controlled for. The mean number of the target trial sequences was around 17 (SD 4.69, Min = 5, Max = 26). Please note. The majority of the study sample had more than ten target trial sequences, only six participants had less than ten trial sequences (three participants had eight trial sequences and three participants each had five, six, and seven trial sequences), see Fig. 1 for the distribution. All performance measures were checked for outliers.^1^

To test which account (strategic-evaluation account versus the orienting account) can explain PES, the effect of the error on performance accuracy was tested. The Wilcoxon rank test was used to test the difference between PCA and PEA.

Based on the normality of the variables, Pearson or Spearman correlation tests were used to investigate the association between performance measures (i.e. mean RT and standard deviation (SD) of correct responses, Error rate, and the magnitude of robust post-error slowing), scores on the EFI (total score and its five subscales), the WFIRS (total score and its seven subscales), and GPA. Only the robust measure of PES was used for such analysis since the traditional measure of PES is confounded by global fluctuations in task performance that can create false PES (i.e. either inflated or deflated PES), for review please see, Dutilh et al. (2012b). Because of multiple correlation tests, the Bonferroni correction was applied by multiplying p values by the number of correlations (i.e. 15 for correlations with the PES, Error rate, mean RT and SD of correct responses). Correlation tests were performed to investigate whether subscales within each questionnaire are highly correlated and dependent on each other. Here, *p* values were corrected by multiplying them by 10 for the EFI subscales and by 21 for the WFIRS subscales. Correlations between the subscales of the EFI and those of the WFIRS were tested and p values were corrected by multiplying them by 35. Please note, the presented *p* values in the following results are corrected.

## Results

The essential performance and scales outcomes are systematically reported in Table 1. Table 1 presents population parameters that include mean, standard deviation, and skewness for all of the questionnaire scores, reaction time measures (mean RT and SD of correct responses, and Error rate), robust and traditional measures of PES, post-error accuracy rate (PEA), and post-correct accuracy rate (PCA).

The Shapiro–Wilk test showed normal distribution of the robust measure of PES (*S*–*W* = 0.979, *df* = 85, *p* = 0.186), the total score on the EFI (*S*–*W* = 0.980, *df* = 85, *p* = 0.220), and GPA scores (*S*–*W* = 0.986, *df* = 56, *p* = 0.753); while, the total score on the WFIRS, Error rate, and PEA were not normally distributed (*p* < 0.004). In line with the outcomes of the Shapiro–Wilk test, Table 1 shows that the total score on the WFIRS and Error rate were positively skewed and PEA was negatively skewed.

### Post-error response adjustments

Participants had an Error rate of 17.21% in average (SD 8.77). The mean reaction time of correct responses and errors were 785 ms (SD 112 ms) and 852 ms (SD 151 ms), respectively. The total number of errors followed by errors was in average 5.93 trial sequences (SD 8.35).

The robust and the traditional measures of PES were significantly different: the mean PES calculated by the traditional measure was significantly lower than that calculated by the robust measure of PES, *t* (84) = − 3.117, *p* = 003 (for the means see Table 1).

Increased variation of RT of correct responses over time was weakly associated with higher error rate (*N* = 85, *r*_*s*_ = 0.29, *p* < 0.006), indicating that fluctuation in RT was related with committing more errors.

The Wilcoxon rank test revealed that the accuracy rate after errors (Mdn = 86.67) was significantly higher than the accuracy rate after correct responses (Mdn = 84.94), *Z *= − 3.22, *p *< 0.001, *r*_s_ = 0.66, indicating that errors slightly but significantly enhanced response accuracy by 1.73%. As mentioned earlier, the testing situation might make participants relatively more oriented to a fast responding with little chance to improve their response accuracy after errors. In this case, a solid estimation of the actual post-error response accuracy improvement a participant could make might be not realized.

So far, only one study associated error monitoring with executive functions (Silver and Goodman 2007) which used reaction times for correct and incorrect responses to index error monitoring. Given that the traditional measures of PES (Dutilh et al. 2012b) and improvement in post-error accuracy are more likely to be confounded, we limited data analyses to the robust measure of PES and questionnaires scores in the subsequent analyses.

### Correlations of the robust measure of PES and RT performance measures with questionnaire scores

Correlations between the magnitude of PES, the total score on the EFI, and GPA scores were not significant (*p* ≥ 0.15). Looking into the correlations between the magnitude of post-error slowing and scores on each scale of the EFI, it appeared that post-error slowing was weakly and positively related with scores on the Strategic Planning scale (*N* = 85, *r* = 33, *p* = 0.03). None of the WFIRS subscales was correlated with post-error slowing (*p* ≥ 0.09).

Correlations between the reaction time measures (namely Error rate, mean RT, and SD of correct responses) and scores on both the EFI and the WFIRS scales were not significant (*p* ≥ 0.18).

### Inter-correlations of the questionnaire scores

The total score on the EFI was significantly associated with the total score on the WFIRS (*N* = 85, *r*_s_ = 66, *p* < 0.001). Table 2 shows correlations between and within the EFI and the WFIRS. As can be seen from Table 2, correlations between scores on the subscales of the WFIRS and scores on the subscales of the EFI (especially for the Organization, the Impulse Control and the Strategic Planning subscales) were moderate and negative.

**Table 2:** Correlation matrix between Scores on Subscales of the EFI and the WFIRS

Self-reported scales	MD	ORG	IC	EM^a^	SP	Family^a^	Work^a^	College^a^	Life-skills^a^	Self-concept^a^	Social functioning^a^
ORG	− 0.11										
IC	− 0.37**	0.51**									
EM^a^	0.07	0.01	0.11								
SP	0.07	0.48**	0.35*	0.24							
Family^a^	0.10	− 0.40**	− 0.40**	− 0.07	− 0.27						
Work^a^	0.02	− 0.41**	− 0.26	− 0.07	− 0.32	0.22					
College^a^	− 0.10	− 0.55**	− 0.41**	− 0.29	− 0.66**	0.41**	0.46**				
Life-Skills^a^	− 0.03	− 0.55**	− 0.42**	− 0.08	− 0.54**	0.53**	0.48**	0.61**			
Self-concept^a^	− 0.31	− 0.38**	− 0.08	0.06	− 0.28	0.38**	0.19	0.46**	0.52**		
Social Functioning^a^	− 0.07	− 0.53**	− .34^*t*^	− 0.17	− 0.39**	0.51**	0.39**	0.52**	0.59**	0.53**	
Risk^a^	0.19	− 0.47**	− 0.57**	− 0.22	− 0.48**	0.61**	0.39**	0.56**	0.65**	0.33	0.53**

The subscales of the EFI are: *MD* motivational drive, *ORG* organization, *IC* impulse control, *EM* empathy, *SP* strategic planning. The subscales of the WFIRS are: family; work; college; life skills; self-concept; social functioning

**p ≤ 0.005, *p ≤ 0.01, ^t^*p *< 0.05, the number of participants for all correlations was 85, except for correlations with the GPA it was 56. ^a^For these variables, Spearman correlation tests were performed. For all other variables Pearson correlation tests were performed

Correlations within each scale were weak to moderate: correlation coefficients ranged from 0.07 to 0.51 between the EFI subscales, and from 0.19 to 0.65 between the WFIRS subscales. This indicates that subscales in each scale are independent from each other, and can be discussed separately.

## Discussion

The main goal of the present study was to explore the association between error monitoring as measured by post-error slowing (PES) during a laboratory test and daily life functioning. It has been argued that during a laboratory test poor ability to monitor ourselves for errors and to adjust our behaviors accordingly may manifest themselves, to some extent, in different life outcomes. Before elucidating the outcomes of testing such relationships, some methodological considerations of PES measures and post-error accuracy will be discussed. The present study showed that the traditional measure of PES was significantly lower than the robust measures of PES. The traditional measure of PES did not count for fluctuations in RT across trials, leading to a diminished magnitude of PES (Dutilh et al. 2012b); and therefore being less reliable in measuring actual individual’s post-error reactivity. Fluctuations in RT cannot be explained by a response strategy; wherein, participants speed up their responding after a correct response until they respond too quickly and make an error (Brewer and Smith 1984) because in the present study RT of errors was slower than RT of correct responses. Alternatively, our RT data is in line with another response strategy; wherein, participants set a deadline for themselves to give an appropriate motor response. Here, once this deadline is passed they might give careless (and likely incorrect) responses (Mohamed et al. 2016). PES, in this case, may reflect an extended deadline to increase the time needed for thoughtful information processing and consequently more accurate responses.

A number of previous studies have shown that response-to-stimulus interval and error rate are critical experimental variables that determine the size and reliability of the PES phenomenon. Here, the magnitude of PES decreases during long response-to-stimulus intervals and increases during short intervals; while, increased post-error accuracy is often found in studies that use relatively long inter-stimulus intervals and decreased post-error accuracy is often found in studies that use relatively short inter-stimulus intervals (see Danielmeier and Ullsperger 2011). A study by Steinborn et al. (2012) is of interest here, as it is one of the few studies that examined PES within a response-stimulus interval of 50 ms. They found a pronounced size of PES and post-error accuracy decrease, supporting the orientation account of PES. The results of the present study are partly consistent with the above-mentioned studies. In this regard, the magnitude of PES was small, especially for the traditional measures of PES, during our lexical decision task with a relatively long inter-stimulus-interval (i.e. 4000 ms). However, the results did not show a decrease in post-error accuracy. It is important to note here that the magnitude of PES is often found to be small during tasks with long inter-stimulus-intervals, which has been interpreted as a decay in PES over time that might be caused by slow task timing (Danielmeier and Ullsperger 2011; Balogh and Czobor 2016).

Regarding post-error response improvement, the present results suggest a positive effect of errors on response accuracy, that is to say, errors slightly but significantly improved response accuracy. This finding, together with PES, supports the strategic-evaluation account of PES and converges with previous research suggesting that PES reflects a cognitive control mechanism and error monitoring (Dutilh et al. 2012a; Lavro et al. 2018; Wang et al. 2016). The reason why the improvement in post-error response accuracy was not high could be related to task characteristics, for instance given the fast stimulus presentation of 150 ms together with the absence of feedback on responses might orientate the participants to fast responding (see Christ 1970). This, in turn, gives little chance to improve response accuracy, and thus questions the use of post-error accuracy measures to reflect the actual ability of participants to improve their responses in the present study. In relation to this Steinborn et al. (2018) addressed the psychometric quality of performance measures (i.e. mean RT, Error rate and variability) and found that such errors are usually rare events entailing a skewed data, which results in low test–retest correlations. This limits the use of error scores in correlative relationships with other variables.

Previous research by Steinborn et al. (2012) indicated that individuals who showed low error rates on the serial mental addition and comparison task (SMACT) had a larger PES and lower accuracy after errors compared to those who showed high error rates. This means, in a broad sense, that error rate can determine the size of PES. Whether this true in the present study is unclear: it is hard to see whether error rate in our lexical decision task is low or acceptable (*M* = 17.2, SD 8.8) since there is no clear conclusive cut-off of the acceptable error rate in the literature. Another factor that may determine the size of PES is task instruction, Steinborn et al. (2012) discussed that accuracy-based task instruction motivate participants to perform well and to put larger orientation to errors, which in turn can lead to stronger interference leading to large PES. In the present study, the used instruction did no put solely emphasis on response accuracy and therefore such interference might not be realized.

With regard to the main outcomes of the present study, results showed that the increased magnitude of the robust PES is weakly associated with planning skills, but not with other executive functions that involve emotional components such as motivational drive and impulse control. This finding is in agreement with previous studies, classifying behavioral monitoring as an executive function that requires only cognitive information processing and planning without emotional involvement (Zimmerman et al. 2016). However, the present finding does not provide arguments against the notion that error monitoring is influenced by emotion regulation during the test. Said differently, when an individual makes an error he/she might feel sad, angry, or anxious. With this in mind, recent studies have indicated that better ability to respond adaptively to errors associates with greater control over negative emotions towards failures in everyday life (Compton et al. 2008; Compton et al. 2011; Potts et al. 2006). Experimentally, some studies have shown that performance indices of low error monitoring can predict poor executive functions (see Robinson et al. 2010; Silver and Goodman 2007; Silver et al. 2006), the present study suggests that might not be the case for executive functions in daily life context.

The present study suggests that increased PES (as a measure of error monitoring) is not associated with functional problems in daily life activities. The study showed that in the student population the magnitude of PES was not associated with poor academic performance assessed by an objective measure (i.e. GPA scores) or a subjective self-reported scale (i.e. the College scale of the WFIRS). This does not replicate previous findings on the association between academic achievement and error monitoring (Fisher et al. 2009; Grammer et al. 2014; Hirsh and Inzlicht 2010; Schumaker et al. 1981). One possible explanation could be that most studies used event-related potentials (specifically error-related Negativity) to measure error monitoring at brain activity level while we use a performance behavioral measure (i.e. PES). In addition, it is still unclear whether there is a causal link between PES and error-related negativity (see Gehring et al. 2018).

Concerning the RT measures (i.e. Error rate, mean RT and SD of correct responses), none of them was correlated with daily functional problems. The associations were also absent for the executive functions, this could be explained by the fact that RT measures, in our task, reflect a variety of cognitive processes such as sustained attention and lexical recognition that are not specific to executive functions, while PES is relatively more specific to executive functions especially planning. From the neurocognitive perspective, some studies have indicated that both PES and executive functioning share, in common, the activity of the same brain area, namely the posterior medial frontal cortex. That is to say, posterior medial frontal cortex dysfunctions can lead to deficits in both executive functions and monitoring ongoing actions, including post-error response adjustments (Danielmeier et al. 2011; Oliveira et al. 2014; Ridderinkhof et al. 2004).

Efficient executive functions measured by the EFI, in particular, those requiring organizational skills, impulse control, planning and the use of strategies, are associated with better daily life functioning as measured by the WFIRS, especially at college settings. In this regard, dynamic environments such as college require daily life adaptive processes, such as flexible behavior modifications, and executive functions. Problems in adaptive processes linked to negative daily life outcomes might be due to less effective engagement of top-down cognitive control over ongoing behaviors (Hirsh and Inzlicht 2010). High executive functioning might enhance learner-directed processes towards effective academic skills and better self-regulation (Petersen et al. 2006), resulting in less problems in daily life and in college. Another explanation for the association between executive functions and daily life could be attributed to the nature of the scales: Both scales have the same nature (namely self-rating scales) that reflect participants’ subjective experiences, increasing the chance of getting more consistent responses between the scales than if they are compared with task performance measures. In a review by Toplak et al. (2013), it has been concluded that performance and self-report scales measures of executive functions tap different underlying mental constructs. This may explain, to a certain extent, the correlations between daily life functioning and executive functioning and the absence of correlations between daily life and PES measures. However, high correlations among self-reported scales could represent difficulties in finding the meaningful differences between the scales. In our study though, the correlations between the EFI and the WFIRS scales were moderate, indicating that these scales are rather independent from each other and have meaningful differences. The same holds for the associations between subscales within each scale.

It is important to emphasize at this point the importance of addressing the association between outcomes derived from controlled experimental environment and those derived from natural behaviors in the real world (Chaytor and Schmitter-Edgecombe 2003; Tupper and Cicerone 1990). The literature review of Chaytor and Schmitter-Edgecombe (2003), suggests that the ecological validity does not apply to the scale itself, but to the inferences that can be drawn from this scale in real life (Chaytor and Schmitter-Edgecombe, 2003; Franzen and Arnett 1997). Typically, the ecological validity has been studied by correlating scores on self-reported scales with traditional parameters of laboratory tasks such as mean reaction time, reaction time variability, and/or a number of errors (Chaytor and Schmitter-Edgecombe 2003; Lamberts et al. 2010). So far, the literature shows weak or no relation between daily life and laboratory measures of executive functions (Adjorlolo 2016), questioning the ecological validity of laboratory tests. In line with the literature, the present study addressed a rarely used laboratory measure in ecological validity studies, namely PES as a measure of task-related error monitoring, and PES showed only a weak association with planning, but not with other measures of executive functioning and real world behaviors. Koerts et al. (2011) discussed that experimental measures are more structured and measure one aspect of cognition; while daily life situations are less structured and more influenced by the cooperation between different cognitive abilities. Indeed, in the present study, no association between error monitoring and daily life functioning was found when it was broadly measured via the WFIRS, but when it was measured in a more structured way via the EFI, PES showed a weak association with planning. This finding might also be due to the nature of the EFI and the WFIRS scales, the scales were not designed to detect precisely the daily problems that are directly linked with poor error monitoring. Instead, the associations reflect, in broad term, indirect relationships between error monitoring and executing dysfunctions. Regardless of the reasons for limited ecological validity, it could be important to know which daily life behaviors are more or less related with PES as an experimental measure of error monitoring, as it may guide intervention or training plan to improve the particular type of executive function skills and during particular settings.

### Limitations

The robust PES in the present study was measured as the reaction time difference between pre-error and post-error responses for trial sequences, wherein the two pre-error trials and the post-error trial were correct. If the second trial after the error is incorrect, one might observe pre-error speeding in the first trial after the error (see Dudschig and Jentzsch 2009), which in turn might corrupt the PES measurement by diminishing the PES. Future studies might control for pre-error speeding by selecting only correct responses of the second trails after the error.

Although the lexical decision task has been used many times to study error processing and related response adjustments in normal and clinical populations, the task does not explicitly reflect specific aspects of everyday performance monitoring. This might limit generalizing the present outcomes. The main goal of using a lexical decision task was to measure individual potentials to adapt and monitor performance and to test whether these potentials are directly associated with daily functioning. As most of the laboratory tasks classically used in performance monitoring research, the present task may reflect action slips besides linguistic errors. In addition, poor performance monitoring on lexical decision tasks was found in a number of clinical or psychiatric disorders such as Parkinson’s disease and ADHD. Furthermore, task performance requires a number of cognitive abilities such as encoding stimuli (strings of letters), searching in memory, and rapid decision-making needed for everyday life. This may explain why performance on the lexical decision task is associated with IQ test (McKoon and Ratcliff 2016).

Another limitation is the use of university students, which could be said to be a relatively high functioning sample compared to the adult population. This may have decreased the variability in task measures and questionnaire scores, limiting the generalizability of the findings. As such, no direct implications for clinical settings can be drawn from the present findings.

Finally, we did not use a psychometric instrument to assess subjective state in performance settings. It can be argued that, within the context of individual-differences research on performance, it is essential to obtain pretest and posttest assessments of the subjective state in performance settings such as distress, worry, and task engagement. As a short outlook, participants’ subjective state may negatively/positively influence their performance and therefore, conclusions about “pure” cognitive skills inferred from performance (such as error monitoring) might be superficially plausible, but actually mistaken. To control for this factor, future studies are recommend to use the Dundee Stress State Questionnaire, a widely accepted and well-evaluated measure with good psychometric properties (DSSQ, Matthews et al. 2002, see also Langner et al. 2010a, b, for methodical aspects of assessing task-induced effects on engagement/distress). DSSQ is a theory-oriented instrument aimed to assess the fundamental dimensions of subjective state in performance settings, namely task engagement, distress, and worry, which can further be divided into more specific sub-facets (e.g. EA as sub-facet of task engagement, TA as sub-facet of distress).

## Conclusion

The laboratory measurement of error monitoring (i.e. post-error slowing) is weakly associated with planning and not with daily life functioning. Findings suggest that post-error slowing may partly be an experimental marker of the active executive processes and that post-error slowing has low ecological validity (as traditional RT measures) and no associations with functioning in a naturalistic environment.

It might be concluded that when daily life functioning is broadly defined, no association with post-error slowing is expected, but when it is narrowly defined in a more structured way, associations might be present.

## Footnotes

1 Regarding detecting outliers of performance measures, the authors examined extreme values within each performance measure. For this purpose, we used visual tools (such as histogram and box plots) followed by a quantitative detection. Any single observation above or below ± 3 standard deviations was identified as an outlier. Three outliers were identified in performance measures: One outlier in the robust measure of PES, one outlier in the traditional measure of PES and the third outlier was identified in three performance measures, namely error rate, post-correct response accuracy, and post-error response accuracy. Visual inspection showed that, specifically the outlier data points in the two PES measures were very close to other data points within the normal distribution. First, the authors checked whether outliers were due to incorrect data entry, incorrect calculation of the performance measures, and/or problems in the testing situation and data collection, which was not the case. Next, data analyses were performed with and without the outliers: removing the outliers had little impact on the study outcomes. Therefore, all data points including outliers were used.
